# Quantifying the mapping precision of genome-wide association studies using whole-genome sequencing data

**DOI:** 10.1186/s13059-017-1216-0

**Published:** 2017-05-16

**Authors:** Yang Wu, Zhili Zheng, Peter M. Visscher, Jian Yang

**Affiliations:** 1 Institute for Molecular Bioscience, The University of Queensland, Brisbane, QLD 4072 Australia; 2 Queensland Brain Institute, The University of Queensland, Brisbane, QLD 4072 Australia; 3 The Eye Hospital, School of Ophthalmology & Optometry, Wenzhou Medical University, Wenzhou, Zhejiang 325027 China

**Keywords:** Genome-wide association studies, Mapping precision, False positive rate, Whole genome sequencing, Imputation

## Abstract

**Background:**

Understanding the mapping precision of genome-wide association studies (GWAS), that is the physical distances between the top associated single-nucleotide polymorphisms (SNPs) and the causal variants, is essential to design fine-mapping experiments for complex traits and diseases.

**Results:**

Using simulations based on whole-genome sequencing (WGS) data from 3642 unrelated individuals of European descent, we show that the association signals at rare causal variants (minor allele frequency ≤ 0.01) are very unlikely to be mapped to common variants in GWAS using either WGS data or imputed data and vice versa. We predict that at least 80% of the common variants identified from published GWAS using imputed data are within 33.5 Kbp of the causal variants, a resolution that is comparable with that using WGS data. Mapping precision at these loci will improve with increasing sample sizes of GWAS in the future. For rare variants, the mapping precision of GWAS using WGS data is extremely high, suggesting WGS is an efficient strategy to detect and fine-map rare variants simultaneously. We further assess the mapping precision by linkage disequilibrium between GWAS hits and causal variants and develop an online tool (gwasMP) to query our results with different thresholds of physical distance and/or linkage disequilibrium (http://cnsgenomics.com/shiny/gwasMP).

**Conclusions:**

Our findings provide a benchmark to inform future design and development of fine-mapping experiments and technologies to pinpoint the causal variants at GWAS loci.

**Electronic supplementary material:**

The online version of this article (doi:10.1186/s13059-017-1216-0) contains supplementary material, which is available to authorized users.

## Background

Genome-wide association studies (GWAS) facilitated by high-throughput genotyping technologies have identified thousands of genetic loci associated with complex traits and diseases in humans [1]. The causal variants and the underlying molecular mechanisms, however, are largely unknown. This is mainly because of the extremely fast pace of GWAS with increasingly large sample sizes and the relative lag of follow-up functional studies of the GWAS loci. There are a few studies that have been able to pinpoint the causal variant and/or the functional gene(s) at a GWAS locus [2–5]. These examples, however, are rare to date, and high-throughput experiments and technologies are in high demand to fine-map the causal variants and/or genes at the GWAS loci [6]. Understanding the distribution of the distances between the top associated variants in GWAS and the underlying causal variants is essential to design and develop such fine-mapping experiments and technologies. In this study, we seek to quantify the empirical distribution of physical distances between GWAS hits and causal variants for different genotyping strategies using simulations.

## Results

The simulations were based on whole-genome sequencing (WGS) data on 3642 unrelated individuals and ~17.6 million genetic variants from the UK10K project [7] after quality controls (QC) (see “Methods”). In each simulation replicate, we randomly sampled a sequence variant as causal variant to generate a phenotype (denoted as *y*) and performed genome-wide association analyses of the simulated phenotype using genotype data from four different genotyping/imputation strategies (see “Methods”): (1) WGS data; (2) SNP-array data imputed to HapMap phase 2 [8] (HapMap2); (3) SNP-array data imputed to 1000 Genomes Project [9] (1KGP) phase 1 (1KGP1); (4) SNP-array data imputed to 1KGP phase 3 (1KGP3). We employed the method described in Yang et al. [10] to mimic the process of SNP-array genotyping followed by imputation using the UK10K-WGS data. That is, we extracted the variants on an Illumina CoreExome array (312,264 SNPs after QC) from the UK10K-WGS data and imputed the UK10K “array data” to HapMap2, 1KGP1, and 1KGP3 using IMPUTE2 [11]. The HapMap2 and 1KGP imputations were performed using the cosmopolitan panels. Note that we did not include the HapMap2-imputed data in the analyses of rare variants because the HapMap2 project was mainly focused on common variants [8]. We also did not perform imputation to the Haplotype Reference Consortium (HRC) [12] because UK10K-WGS is part of HRC (see below for HRC-imputation based on genotyped data from an independent cohort). The number of variants for each genotyping strategy is listed in Additional file 1: Table S1. We repeated the simulation 50,000 times for common (minor allele frequency, MAF > 0.01) and rare (0.0003 < MAF ≤ 0.01) variants, respectively, and selected the top associated variant at a genome-wide significance level from each GWAS analysis.

Before conducting the analysis to quantify mapping precision (i.e. physical distance between the top associated variant in GWAS and the actual causal variant), we calibrated the genome-wide false positive rate (GWFPR, the number of simulations with at least one false positive divided by the total number of simulations) under the null hypothesis (see “Methods”), where the phenotypes were generated from a standard normal distribution without any genetic effect. We conducted the simulation with 1000 replicates, and calculated the GWFPR (also known as family-wise error rate [FWER]) at a range of threshold *P* values (from 5e-8 to 1e-11). We found that rare variant association was extremely sensitive to the skewness of the phenotype distribution as demonstrated by the highly inflated test-statistics in GWAS for *y* ^2^ (Additional file 1: Figure S1). We therefore performed a rank-based inverse-normal transformation (INF) of the phenotypes in all the subsequent analyses. Under the null hypothesis, GWFPR at *P* < 5e-8 was smaller than 0.05 for HapMap2-based imputation (Additional file 1: Figure S2), suggesting that the GWFPR was well controlled in most published GWAS based on SNP genotyping arrays or HapMap2-based imputation. For GWAS using WGS or imputed WGS data, however, 5e-8 seems inadequate to control the GWFPR at 0.05 (GWFPR = 0.34 for WGS or 1KGP3-imputed data) (Additional file 1: Figure S2), consistent with the result from a previous study [13]. The inflation of GWFPR for imputed data was not due to the inclusion of SNPs with low imputation INFO score (Additional file 1: Figure S3). There is no inflation in test-statistics (Additional file 1: Table S2), implying that the inflated GWFPR is due to the number of independent tests being larger than 1 million. The threshold *P* value at GWFPR = 0.05 needs to be somewhere between 5e-8 and 1e-8 for common variants and close to 5e-9 for all variants in the UK10K-WGS or 1KGP3-imputed data. We therefore recommend to use a threshold of 1e-8 for GWAS with common variants, which might be slightly conservative for current datasets but should be appropriate for data from WGS or imputation-based studies in the future because the number of variants is expected to increase with the increase of sample size [14] and improved genome coverage. For GWAS using all the genetic variants (including rare), we recommend to use a threshold of 5e-9 for current datasets and a more stringent threshold (e.g. 1e-9) for data in the future with larger sample size and higher coverage. In addition, we also strongly recommend to perform an INF of the phenotype for rare variant associations given the highly inflated GWFPR for phenotypes of skewed distribution under both the null (Additional file 1: Figure S1) and alternative (Additional file 1: Figure S4) hypotheses. However, there is a caveat that under the alternative hypothesis where there are real genetic effects, the estimated effect sizes for the INF-transformed phenotype will be slightly smaller than those for the original phenotype.

**Fig. 1:**
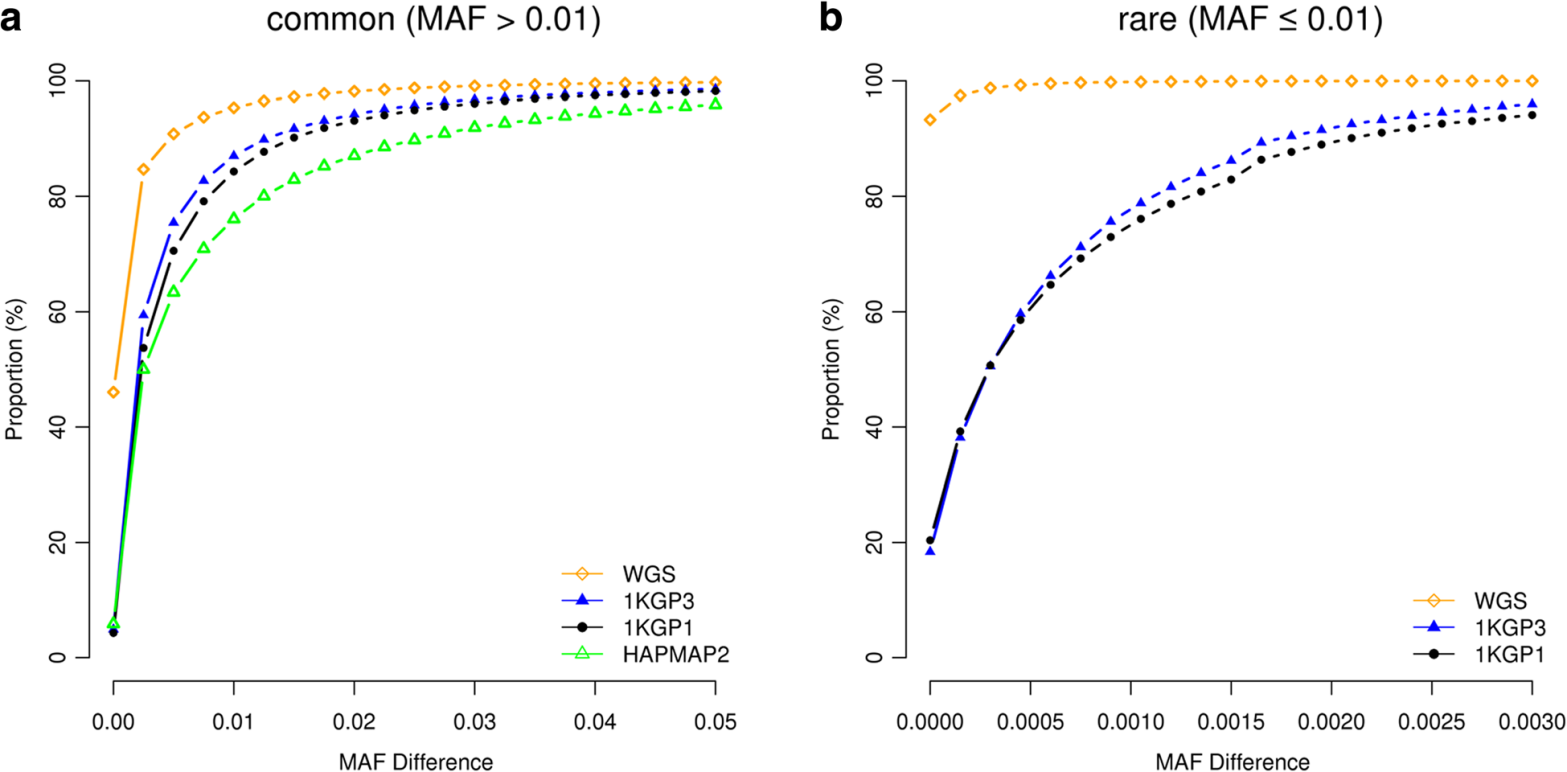
Differences in MAF between GWAS hits and causal variants for different genotyping strategies. Results are from 50,000 simulations based on the UK10K-WGS data for common (**a**) and rare (**b**) causal variants, respectively. Shown on the *y*-axis is the proportion of causal variants that were mapped to variants with MAF differences smaller than a value specified on the *x*-axis

**Fig. 2:**
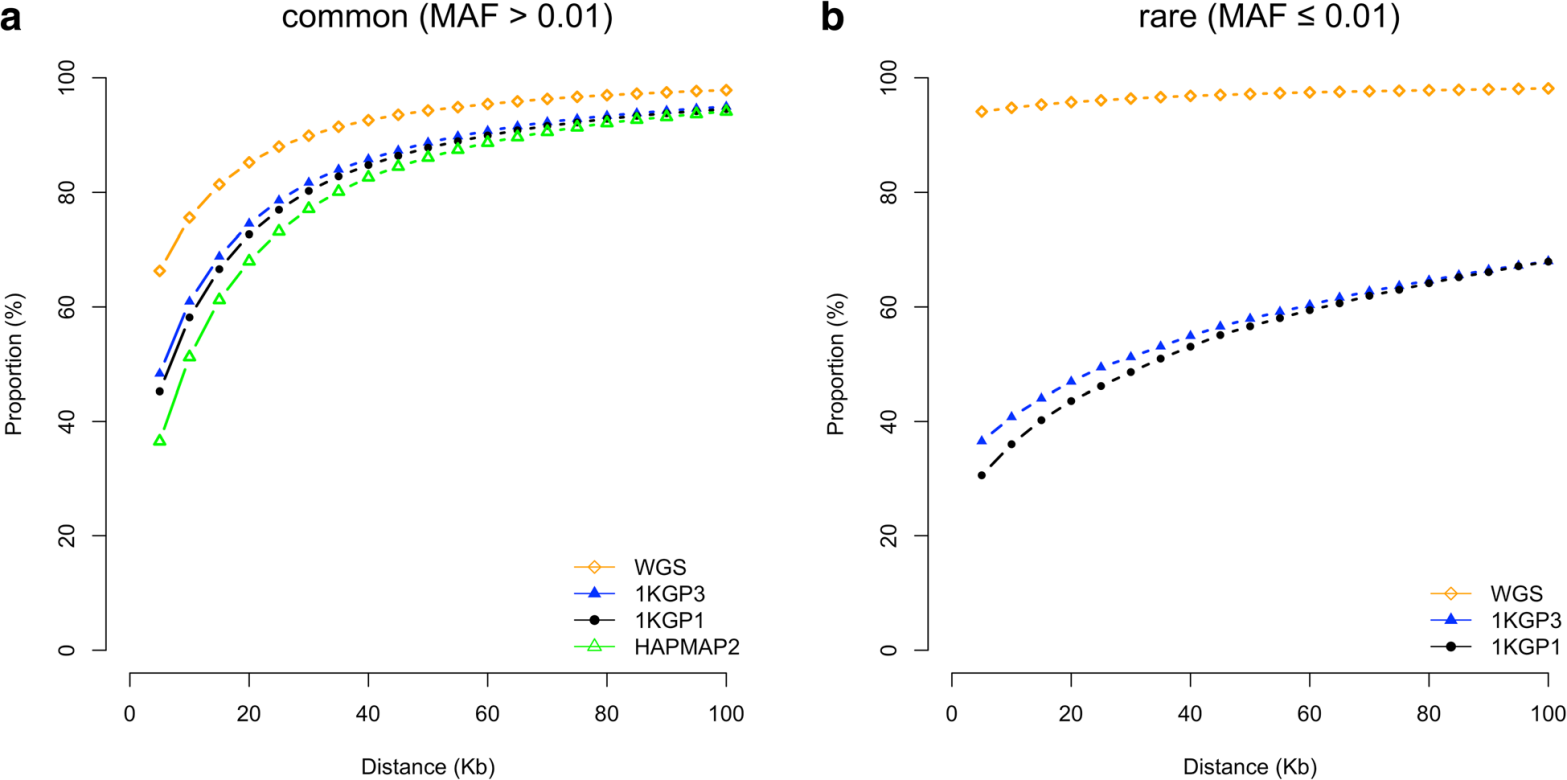
Mapping precision of GWAS based on different genotyping strategies. Results are from 50,000 simulations for causal common (**a**) and rare (**b**) variants, respectively, based on the UK10K-WGS data. Shown on the *y*-axis is the proportion of causal variants that were mapped to variants within a certain distance as specified on the *x*-axis

**Fig. 3:**
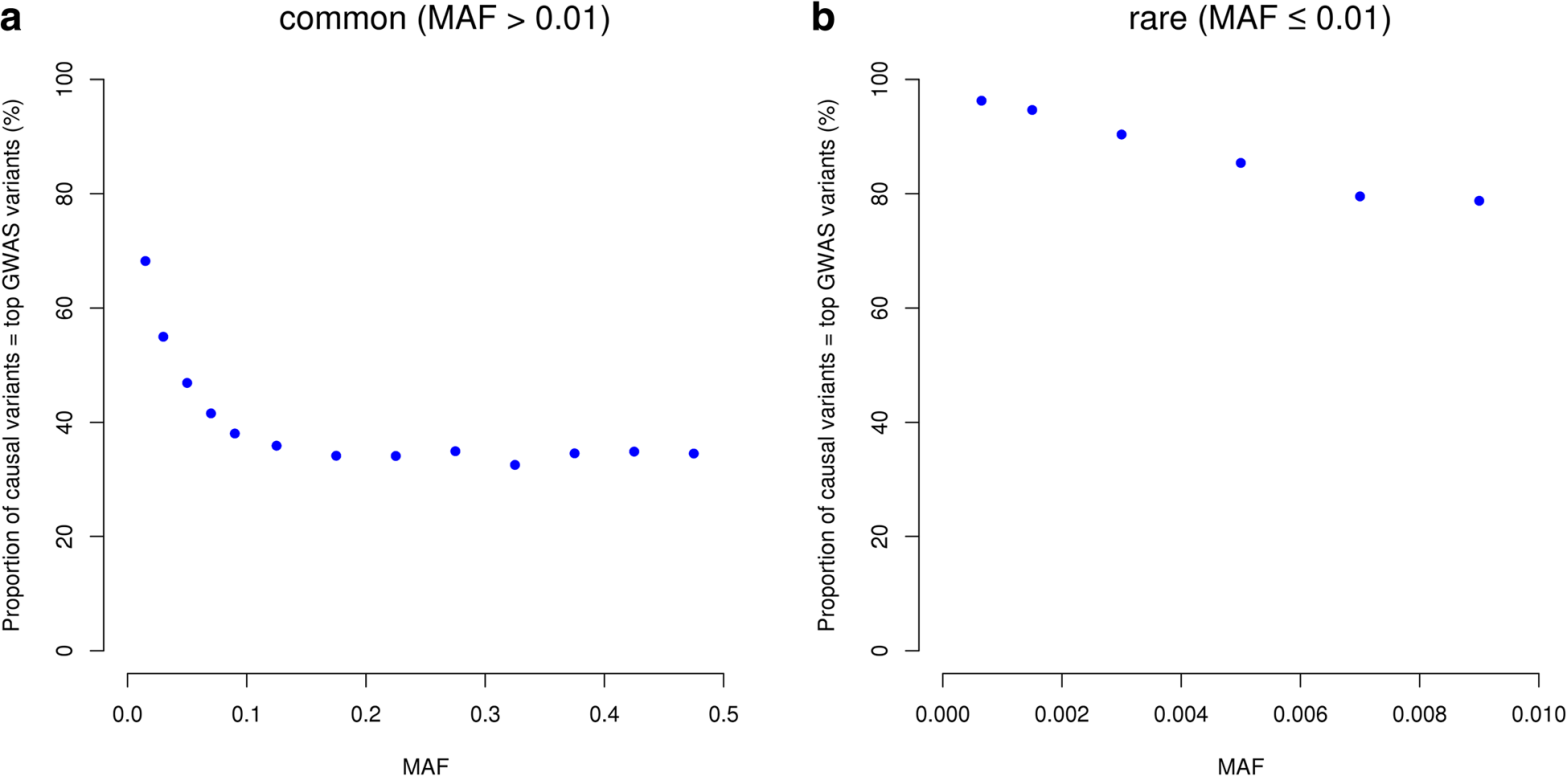
Proportion of causal variants that are the top associated variants in GWAS. Shown are the mean values in MAF bins from 50,000 simulations based on the UK10K-WGS data for common (**a**) and rare (**b**) variants, respectively

Having calibrated above the GWFPR under the null hypothesis, we then turned to quantify the mapping precision under the alternative hypothesis (see “Methods”). Since most of the published GWAS used the *P* value threshold of 5e-8, we performed most analyses based on this threshold (see below for the discussion about the influence of *P* value threshold on mapping precision). The total number of tests involved in the whole simulation process would have been extraordinarily large (100,000 simulations × 17,612,713 variants). To minimize the number of false positives, we limited the number of tests by focusing only on GWAS results in a 20Mbp region centered at the simulated causal variant. The number of GWAS hits identified in all simulations for each genotyping strategy is listed in Additional file 1: Table S3. The result shows that the differences in MAF between GWAS hits and causal variants were very small (at least 95.0% of the common causal variants were mapped to variants with MAF differences < 0.05 and at least 94.6% of the rare causal variants were mapped to variants with MAF differences < 0.003) (Fig. 1), suggesting that the association signal at a rare causal variant is highly unlikely to be mapped to a common variant in GWAS using either WGS or imputed data and vice versa. We then quantified the proportion of GWAS hits within a given physical distance from the corresponding causal variants (Fig. 2). For common variants, the majority of the top associated variants in GWAS were in < 100 Kbp distance from the causal variants, from 94.8% for GWAS using HapMap2-imputed data to 98.3% using WGS data (Fig. 2a), in line with the result from a recent study that most of the candidate causal variants (inferred from a fine mapping analysis with epigenetic data) are within 100 Kbp of the GWAS top hits [15]. It should be noted that the result for WGS data was not 100% because the causal variant was not always the top associated variant in GWAS (Fig. 3) due to the complicated linkage disequilibrium (LD) structure between genetic variants in close proximity and the sampling variation in the test-statistics (see Additional file 1: Figure S5a for a simple example). The results also suggest that for published GWAS using imputed data from HapMap2 or 1KGP, at least 80% of the top associated GWAS variants are within 33.5 Kbp distance of the causal variants. The mapping precision for 1KGP1-based imputation was higher than that for HapMap2-based imputation but the difference was not large (27.6 Kbp versus 33.5 Kbp at 80%). The difference between 1KGP1 and 1KGP3 was subtle (27.6 Kbp versus 25.1 Kbp at 80%). All the results suggest that the strategy of SNP array-based genotyping with subsequent imputation has already provided a high mapping resolution that is comparable with that using WGS, consistent with the conclusion from our previous study [10] that WGS is not a cost-effective approach to map common variants for complex traits.

**Fig. 4:**
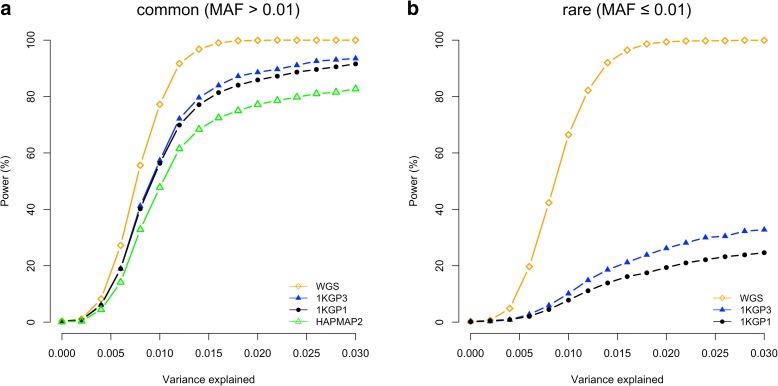
Statistical power of GWAS based on different genotyping strategies. Power is calculated as the proportion of simulations with a least a variant at *P* < 5e-8. Shown are the results from 5000 simulations for common (**a**) and rare (**b**) variants, respectively, at each heritability level

For rare variants, however, the results were different. The difference between WGS and 1KGP-impution was very large. There were 94.2% of the GWAS hits within a distance of 5 Kbp of the causal variants for WGS but only 36.6% for 1KGP3-based imputation. This is because the number of variants in high LD with a rare variant was much smaller than that for a common variant (Additional file 1: Figure S6) and thus it is more likely for a rare causal variant being detected as the top signal in WGS data than a common variant. It is shown in Fig. 3 that 98% of causal variants were detected as the top signals in GWAS for very rare variants (0.0003 < MAF < 0.001) and the proportion decreased to ~30–40% for very common variants (MAF > 0.1). Approximately 68.1% of the GWAS hits were within a distance of 100 Kbp of the causal variants for 1KGP3-based imputation (Fig. 2b), which was much smaller than that (98.2%) for WGS (Fig. 2b). These results suggest that mapping precision of GWAS using imputed data for rare variants is much lower than that for common variants (Fig. 2), and these results are not driven by sampling variation in LD *r* ^2^ (Additional file 1: Figure S7). Moreover, the statistical power of detection for rare variants using imputed data was also much lower than that for common variants (Fig. 4) because rare variants were less well imputed than common variants [12, 16]. There were a substantial proportion of causal variants, especially rare causal variants, which were mapped to variants in more than 100 Kb distance even at an extremely stringent *P* value threshold (i.e. *P* < 5e-11) (Additional file 1: Figure S8). This is because in comparison with common variants, rare variants have fewer LD proxies within 100 Kb distance (Additional file 1: Figure S6), less likely to be present in the reference panel (2.2% of the common variants and 50.4% of the rare variants in UK10K-WGS are absent in 1KGP3), and less well imputed even if they are present in the reference panel [12, 16], their association signals are therefore more likely to be mapped to distant variants due to the complicated LD structure of genome as illustrated in Additional file 1: Figure S9. Taken all together, our results demonstrate the benefit of using WGS as a strategy for detecting and fine-mapping rare variants simultaneously. For real data, ignoring cost considerations, the advantage of using WGS in GWAS depends on the proportion of heritability for the trait or disease that is attributable to rare variants [10, 17]. In addition, the sample size of WGS data needs to be very large because the statistical power of GWAS to detect a variant is determined by the non-centrality parameter (NCP) of the *χ* ^2^ test-statistic, i.e. NCP = *nq* ^2^/(1 – *q* ^2^), where *n* is the sample size of the GWAS data, *q* ^2^ = 2*f*(1 – *f*)*b* ^2^ with *b* being the effect size per allele and *f* being the allele frequency. For rare variants, if *q* ^2^ is small, NCP ≈ *nq* ^2^ ≈ 2*nfb* ^2^.

**Fig. 5:**
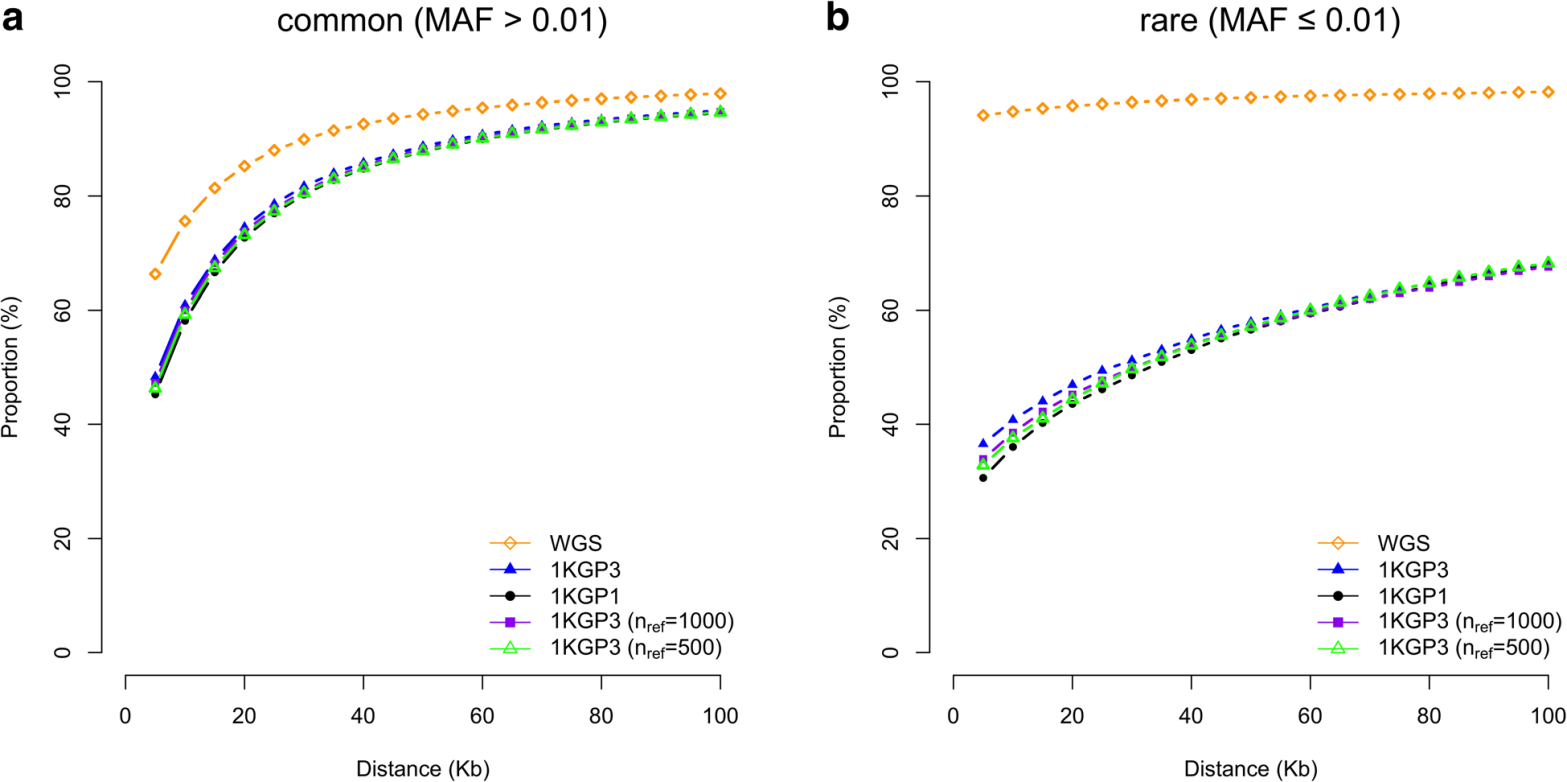
Mapping precision of GWAS based on imputations with different sample sizes of the reference panel. Shown are results from 50,000 simulations for common (**a**) and rare (**b**) variants, respectively. 1KGP3 (*n*_ref_ = 1000) and 1KGP3 (*n*_ref_ = 500): SNP array data imputed to a random subset of 1000 and 500 individuals randomly sampled from 1KGP3, respectively

We observed little difference in mapping precision between the analyses based on data imputed to 1KGP1 and 1KGP3 (Fig. 2) despite that the sample size of 1KGP3 (*n*_ref_ = 2504) is ~2.5 times larger than that of 1KGP1 (*n*_ref_ = 1092). There was an apparent, although also not large, difference in power between 1KGP1 and 1KGP3 (Fig. 4). We then investigated the mapping precision as a function of *n*_ref_ by re-running the imputation to a random subset of individuals from 1KGP3 (*n*_ref_ = 500 and 1000). The additional imputation analyses showed consistent results, i.e. power slightly increased with *n*_ref_ in particular for rare variants (Additional file 1: Figure S10) whereas mapping precision was almost independent from *n*_ref_ for either common or rare variants (Fig. 5). To further investigate the influence of *n*_ref_ on the mapping precision of GWAS using imputed data, we performed additional analyses using genotyped data from a larger GWAS cohort (i.e. the Health Retirement Study [HRS] [18]) and imputed the genotyped data to a much larger reference panel (i.e. HRC). There were 8479 unrelated individuals in HRS genotyped on ~1.7 million SNPs (1,451,882 common and 243,548 rare) after QC [10]. We left out 50,000 common and 50,000 rare SNPs as a pool to sample causal variants for simulations and imputed the genotypes of the remaining SNPs to 1KGP3 and HRC. We performed 50,000 simulations for common and rare variants, respectively. In each simulation replicate, we randomly sampled a variant from the causal variant pool (50,000 common and 50,000 rare SNPs) and simulated a quantitative phenotype using the method described above with *q* ^2^ = 0.87% (NCP = 74, similar as that in the UK10K simulation). We then performed GWAS analyses of the simulated phenotype using the 1KGP3- and HRC-imputed data. We observed little difference in mapping precision between the results using 1KGP3- and HRC-imputed data (Additional file 1: Figure S11), consistent with our observations above that mapping precision of GWAS using imputed data was almost independent of *n*_ref_. We further performed simulations in a subset of HRS individuals (*n* = 3642, the same sample size as UK10K) using the same setting as in the UK10K simulations above. The result remained largely unchanged (Additional file 1: Figure S11). It is interesting to note that mapping precision for rare variants in the 1KGP-imputed HRS data was much higher than that in the 1KGP-imputed UK10K data (Additional file 1: Figure S11 and Fig. 2). This is because almost all the rare causal variants in HRS were available in 1KGP3 (only 5.9% were not available) whereas more than a half (50.2%) of the rare causal variants in UK10K were not available in 1KGP3. To confirm this, we re-calculated the mapping precision in the 1KGP3-imputed UK10K data focusing only on the causal variants that were available in 1KPG3. The result was almost identical to that observed in the HRS data imputed to either 1KGP3 or HRC (Additional file 1: Figure S12). These observations suggest that the low mapping precision for rare variants in GWAS using imputed data is mainly due to a large proportion of rare causal variants that are not available in the reference. Taken together, our results seem to suggest that the mapping precision of GWAS using imputed data increases with the variant-coverage of the imputation reference but is almost independent of the sample size of the reference (although these two factors are intertwined). In addition, we observed that having the causal variants in the reference not only improved mapping precision (Fig. 2 and Additional file 1: Figure S13) but also increased statistical power (Fig. 4 and Additional file 1: Figure S14). The difference in power between the two sets of variants (available versus not available in the reference) can be quantified as the loss of power attributable to imputation accuracies (the variance explained by GWAS hit *q*_*GWAS*_^2^ = *q* ^2^ *R*_*imp*_^2^, where *R*_*imp*_^2^ is the squared imputation accuracy) and imperfect tagging (*q*_*GWAS*_^2^ = *q* ^2^ *R*_*imp*_^2^ *r* ^2^, where *r* ^*2*^ is LD *r* ^*2*^ between GWAS hit and causal variant).

We next investigated the influence of GWAS sample size (*n*) on mapping precision. We demonstrated by simulations under a simple scenario that the probability of causal variant being detected as the top signal in GWAS with sequencing data depends on NCP (Additional file 1: Figure S5), which is a function of both *q* ^2^ and *n* (see the equations above). This explains why the mapping precision slightly decreased with decreased *n* or *q* ^2^ in either WGS or 1KGP-imputed data (Additional file 1: Figure S15). In our simulations, in order to obtain sufficient power to detect the simulated genetic effects at a genome-wide significance level (e.g. *P* < 5e-8) using a relatively small sample size (*n* = 3642 unrelated individuals), we simulated causal variants of relatively large effect (*q* ^2^ = 2% in most of the analyses). Given *n* = 3642 and *q* ^2^ = 2%, the NCP at any of the simulated causal variants was 74.3, which is approximately equivalent to a setting with *n* = 250,000 and *q* ^2^ = 0.03% (note that the estimated mean *q* ^2^ of the published 679 height SNPs is ~0.03% from the GIANT meta-analysis [19] with *n* = ~250,000), suggesting that the conclusions we drew from our simulations can be applied in general to studies at the current scale (*n* = 100,000s) and that mapping precision at the known loci will be improved in the future with larger sample sizes. The conclusion has further been supported by evidence from simulations in the HRS dataset with a wider range of sample sizes and NCP (Additional file 1: Figure S16). In addition, we investigated the impact of *P* value threshold on mapping precision. We found that mapping precision of GWAS using sequenced or imputed common variants or sequenced rare variants did not change with *P* value threshold (Additional file 1: Figure S8). However, mapping precision of GWAS using rare imputed variants at *P* < 5e-11 was substantially larger than that at *P* < 5e-8. This is because distant tagging variants were disproportionately more likely to be removed by the stringent threshold *P* < 5e-11 (Additional file 1: Figure S17).

## Discussion and conclusions

**Fig. 6:**
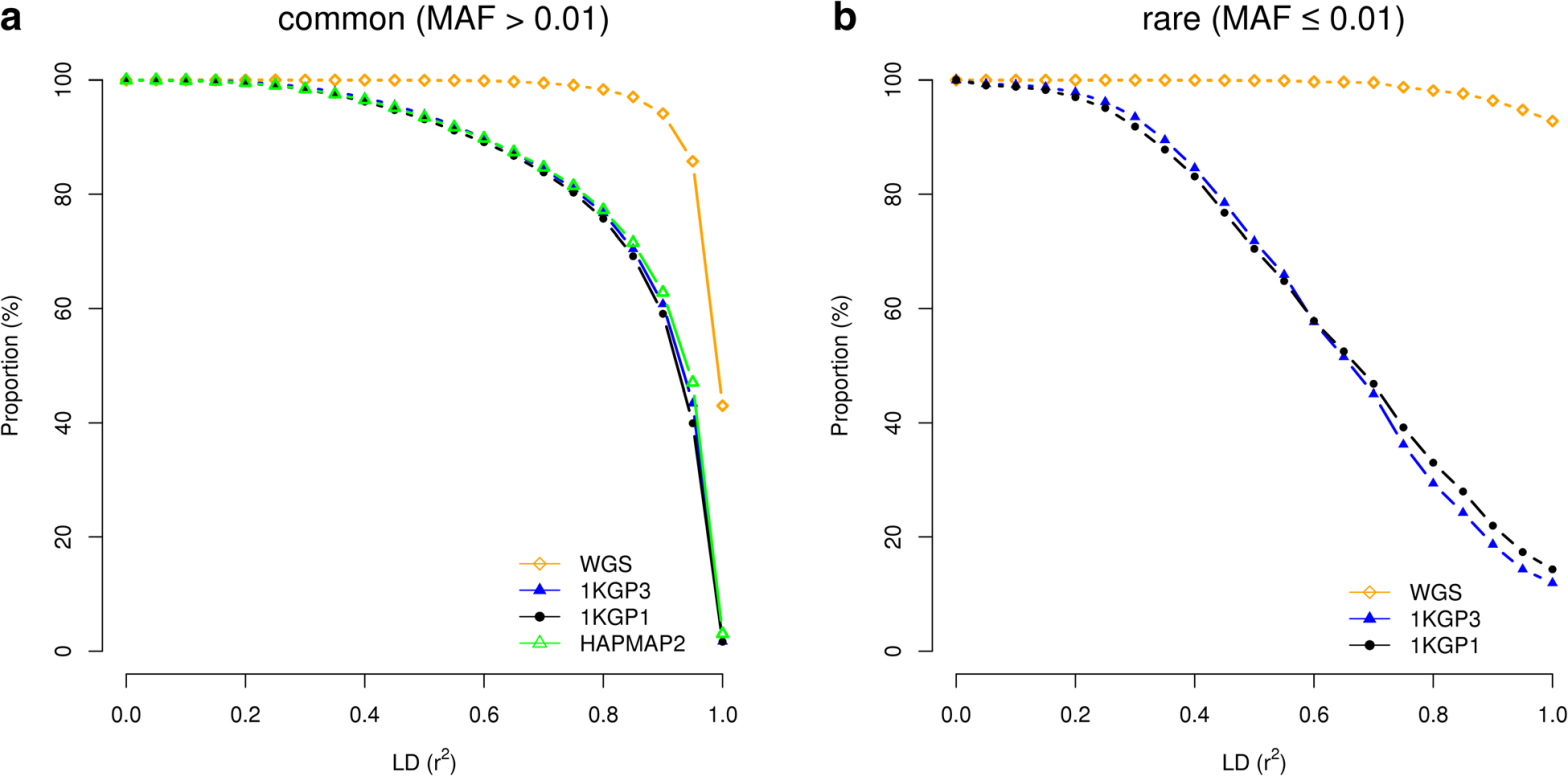
Mapping precision of GWAS as measured by the squared LD correlations between causal variants and GWAS top SNPs based on different genotyping strategies. Results are from 50,000 simulations for causal common (**a**) and rare (**b**) variants, respectively, based on the UK10K-WGS data. Shown on the *y*-axis is the proportion of causal variants that were mapped to variants with LD *r* ^2^ smaller than a certain threshold as specified on the *x*-axis

We have shown above results from simulations where the causal variants were randomly sampled from the sequence variants. In reality, however, it might not be the case. It has been suggested in previous studies that trait-associated or disease-associated variants are not randomly distributed but enriched in some functional categories of the genome such as the DNase I hypersensitive sites (DHSs) [20, 21]. We therefore performed simulations by sampling causal variants from DHSs where the SNPs are in lower LD [10, 20]. The results were almost exactly the same as those presented above (Additional file 1: Figure S18), suggesting mapping precision is almost independent of the distribution of the causal variants in the genome. All the imputation analyses presented above are based on Illumina CoreExome array. We chose the Illumina CoreExome array because it is the most cost-effective SNP array with respect to capturing genetic variation among all the SNP arrays investigated in a previous study [10] and because the number of SNPs on an Illumina CoreExome array (312,264 SNPs after QC) is relatively small (Additional file 1: Table S4) so that the mapping precision quantified based on this array is likely to be conservative and can therefore be used as a benchmark to guide the design of fine-mapping studies. In a meta-analysis of GWAS, however, data from different participating cohorts are usually genotyped on different types of SNP genotyping arrays. We then repeated the analysis for four additional types of SNP arrays (i.e. Affymetrix 6, Affymetrix Axiom Genome-Wide EUR Array [22], Illumina OmniExpress, and Illumina Omni2.5). The number of variants in each array is listed in Additional file 1: Table S4. The results were all very similar except that Illumina Omni2.5 performed slightly better than the other types of arrays for both common and rare variants (Additional file 1: Figure S19), which is likely because of its denser SNP coverage. Given these results, if data from all participating cohorts are imputed to the same imputation reference (e.g. 1KGP), heterogeneity in mapping precision across cohorts is likely to be small. These results also imply that to design a SNP-array based GWAS study with a fixed budget, the most cost-effective design is to choose the cheapest SNP array with genome-wide coverage and maximize experimental sample size, in line with the conclusion drawn from our previous study [10]. In all the analyses above, we used physical distance to assess the mapping precision. In practice, however, it is sometimes also useful to know the distribution of LD between GWAS top hits and causal variants. To this end, we quantified the mapping precision by the squared LD correlation (*r* ^2^) between causal variants and GWAS hits (Fig. 6). Interestingly, for common variants in GWAS using imputed data, at least 77.3% of the association signals were mapped to SNPs in LD *r* ^*2*^ > 0.8 with the causal variants. We further developed an online tool (gwasMP) [26] for querying our results with different thresholds of physical distance and/or LD (http://cnsgenomics.com/shiny/gwasMP).

There are certainly more complicated scenarios (e.g. multiple common and rare causal variants in a very small genomic region) that have not been investigated in our simulations. However, these scenarios are unlikely to be the norm and thus are unlikely to bias our results substantially. With limited sample size of the WGS data (*n* = 3642) we were only able to quantify the mapping precision for rare variants with MAF down to 0.0003. For rarer variants, larger population-based cohorts with WGS data are required. Our conclusions were drawn from simulations based on modern SNP arrays with 100,000s SNPs, which cannot be applied to studies based on low-dense markers. It should also be noted that all our results are from analyses in European populations, these results need to be applied with caution to non-European populations (e.g. Asian and African populations), given the substantial differences in LD structure between Europeans and non-Europeans. We also did not simulate a case-control design because the sample size of UK10K-WGS data is not large enough to simulate an ascertained case-control study of sufficiently large sample size for a disease of reasonable prevalence. However, the general conclusions about mapping precision should be applicable to case-control studies because mapping precision is essentially determined by the strength of the association signal, LD structure and imputation precision, rather than the scale of the phenotype. Nevertheless, this needs to be confirmed in the future by simulations of case-control design using large WGS datasets.

In summary, we performed simulations based on WGS data to quantify the mapping precision for complex traits and diseases under a number of different scenarios. The results show that SNP array-based genotyping with subsequent imputation to any of the commonly used reference panels has provided very high mapping precision for common variants. We predict that at least 80% of the top associated common variants identified from published GWAS are within 33.5 Kbp distance of the causal variants, and mapping precision at these loci can be improved in the future with larger sample sizes. For imputed data, the differences in mapping precision between different SNP genotyping arrays were trivial. Mapping precision of GWAS using imputed data increased with variant-coverage of the reference panel but was almost independent of sample size of the reference. These two factors, however, are not independent. WGS with increasingly large sample sizes and improved sequencing technology will provide more genetic variants [14] in the reference panels in a foreseeable near future, which will certainly improve the mapping precision of GWAS using data imputed from these large reference panels. For rare variants, the mapping precision of GWAS based on WGS data was extremely high, much higher than that based on imputation. This implies the potential of using WGS as an efficient strategy for detecting and fine-mapping rare variants at the same time. All these findings provide an important benchmark to inform the design and development of fine-mapping experiments and technologies in the future to identify causal variants at the GWAS loci.

## Methods

### Simulation based on WGS data

We used WGS data from the UK10K project (UK10K-WGS) [7] for simulations. The data consist of 3781 individuals and ~45.5 million genetic variants. We excluded SNPs with missingness > 0.05, Hardy-Weinberg equilibrium test *P* value < 1 × 10^–6^, or minor allele count (MAC) < 3 (equivalent to MAF < 0.0003) using PLINK [23]. We chose a MAC threshold of 3 because we sought to choose a MAF threshold as low as possible to make general inferences about rare-variant associations but excluded singletons and doubletons as they are more subject to sequencing errors. We further removed individuals with genotype missingness rate > 0.05 and one of each pair of individuals with estimated genetic relatedness > 0.05. The genetic relatedness was estimated from GCTA [24] using all the common SNPs on HapMap phase 3 (HapMap3). A total of 3642 unrelated individuals and 17.6 million variants were retained for analysis. We randomly sampled a variant from UK10K-WGS as causal variant and generated the phenotype based on the model *y* = *g* + *e*, with *g* = *wu* and $w=\left( x-2 f\right)/\sqrt{2 f\left(1- f\right)}$, where *x* is the indicator variable for the genotypes of causal variant (coded as 0, 1or 2), *f* is the frequency of the coded allele, and *u* is the effect size per standardized genotype sampled from *N*(0, 1). The residual *e* was generated from *N*(0, *var*(*g*)(1/*q* ^2^ − 1)) with *q* ^2^ being the proportion of variance in phenotype explained by the causal variant. We performed a GWAS analysis for the simulated trait using the variants from different genotyping strategies (see below for details about the genotyping strategies) and selected the top associated variant that passed a genome-wide significance level (e.g. *P* value < 5e-8). We repeated the simulation to quantify the power at different levels of *q* ^2^ (from 0 to 3% at 0.2% intervals; 5000 replicates at each *q* ^2^ level). Note that the simulations at *q* ^2^ = 0 quantify the false positive rate. We then repeated the simulation 50,000 times at *q* ^2^ = 2% to quantify the mapping precision (i.e. physical distance between the top associated variant identified in GWAS analysis and the simulated causal variant) for common and rare variants, respectively. We further repeated analysis to quantify the mapping precision by sampling causal variants from the DNase I hypersensitive sites (DHSs) to mimic the observation that genetic variants associated with complex traits are enriched in DHSs [10, 20].

### Imputation of SNP-array data to multiple reference panels

We performed simulations to quantify the mapping precision using three different genotyping strategies, i.e. WGS, SNP-array data imputed to HapMap 2 reference panel (HapMap2) [8], and SNP-array data imputed to 1000 Genome project reference panels (1KGP) [9]. The method to mimic the strategy of SNP-array genotyping followed by imputation is described in Yang et al. [10]. That is, we extracted SNPs that are on Illumina CoreExome arrays from the UK10K-WGS data, phased genotypes using SHAPEIT [25], and imputed the data to HapMap2, 1KGP phase 1 (1KGP1), and 1KGP phase 3 (1KGP3) by IMPUTE2 [11]. To investigate the power and mapping precision as a function of sample size of the imputation reference, we further performed the imputation analyses using a subset of individuals randomly sampled from 1KGP3 (*n* = 500 and 1000) as the reference panel.

To investigate the influence of reference sample size on the mapping precision of GWAS using imputed data, we performed additional analyses using genotyped data from HRS and imputed the genotyped data to HRC. There were 8479 unrelated individuals in HRS genotyped on ~1.7 million SNPs (1,451,882 common and 243,548 rare) after QC. We left out 50,000 common and 50,000 rare SNPs as a pool to sample causal variants for simulations and imputed the genotypes of the remaining SNPs to the 1KGP3 and HRC reference panel [12] respectively using Sanger imputation server (https://imputation.sanger.ac.uk/).

## Additional files

Additional file 1: This PDF file contains **Figures S1–S19,** **Tables S1–S4,** and **Text S1**. (PDF 5824 kb)
