# Supplementary material for: Quantifying the mapping precision of genome-wide association studies using whole-genome sequencing data
Source: Genome Biol. 2017 May 16;18:86. doi: 10.1186/s13059-017-1216-0 (PMC5432979; doi:10.1186/s13059-017-1216-0)
Supplement: Supplementary file 1 — This PDF file contains Figures S1–S19, Tables S1–S4, and Text S1. (PDF 5824 kb) [file 13059_2017_1216_MOESM1_ESM.pdf]

Additional file 1

Quantifying the mapping precision of genome-wide association studies using whole-genome sequencing data

Yang Wu[1,2], Zhili Zheng[3,1], Peter M. Visscher[1,2], Jian Yang[1,2,*]

[1] Institute for Molecular Bioscience, The University of Queensland, Brisbane, Queensland 4072, Australia

[2] Queensland Brain Institute, The University of Queensland, Brisbane, Queensland 4072, Australia

[3] The Eye Hospital, School of Ophthalmology & Optometry, Wenzhou Medical University, Wenzhou, Zhejiang 325027, China

[*] Correspondence: Jian Yang (jian.yang@uq.edu.au)

Figures S1 to S19

Tables S1 to S4

Text S1

Reference

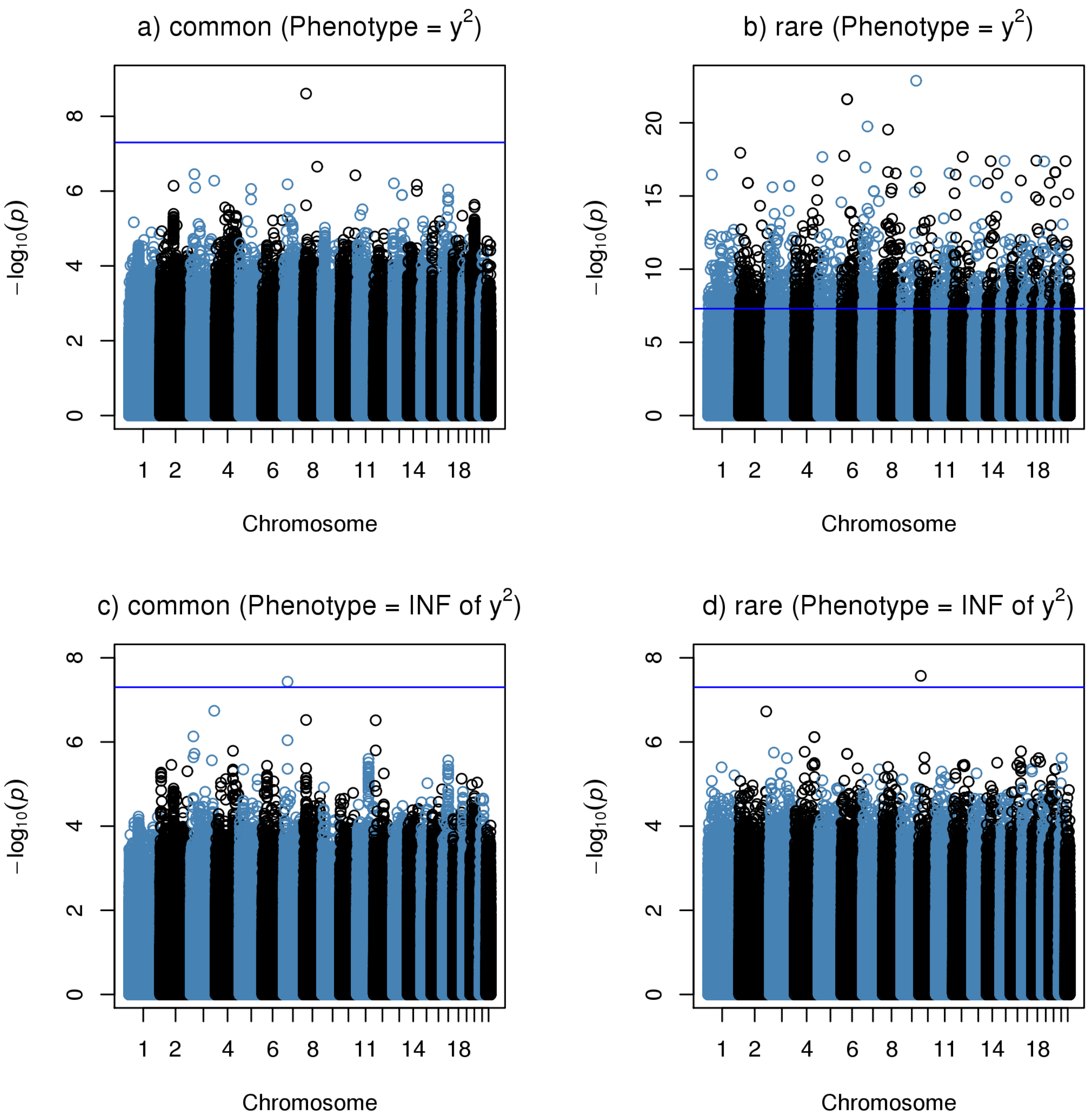

Figure S1 GWAS using WGS data for phenotypes with normal and skewed distributions under the null. The phenotypes (y) were simulated under the null model ($q^2 = 0$), where q^2 is denoted as the proportion of variance in phenotype explained by the causal variant. In panels (a) and (b), the analysis was performed on y^2. Since y is generated from $N(0, 1)$, y^2 follows a chi-squared distribution which is highly skewed. In panels (c) and (d), the phenotypes were adjusted by the rank-based inverse-normal transformation (INF).

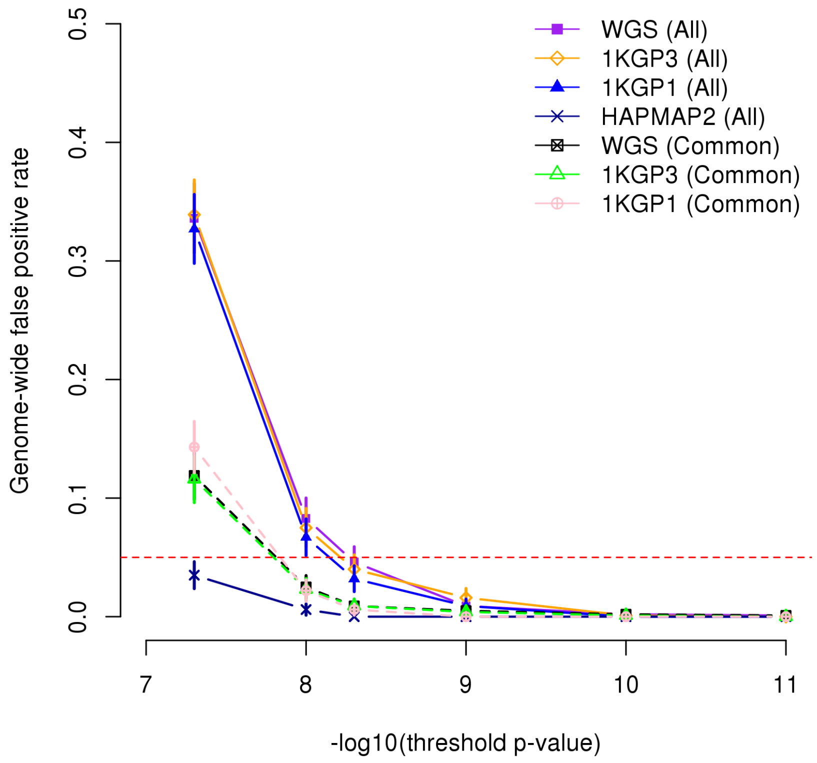

Figure S2 Genome-wide false positive rate for GWAS based on different genotyping strategies. Genome-wide false positive rate (*y*-axis) was calculated as the number of simulations with at least one false positive divided by the total number of simulations at a *P*-value threshold (*x*-axis). Shown are the results and 95% confidence interval from 1,000 simulations based on the UK10K-WGS data under the null hypothesis where the phenotypes are generated from *N*(0,1) without any genetic effect. The red dashed line represents a genome-wide false positive rate of 0.05.

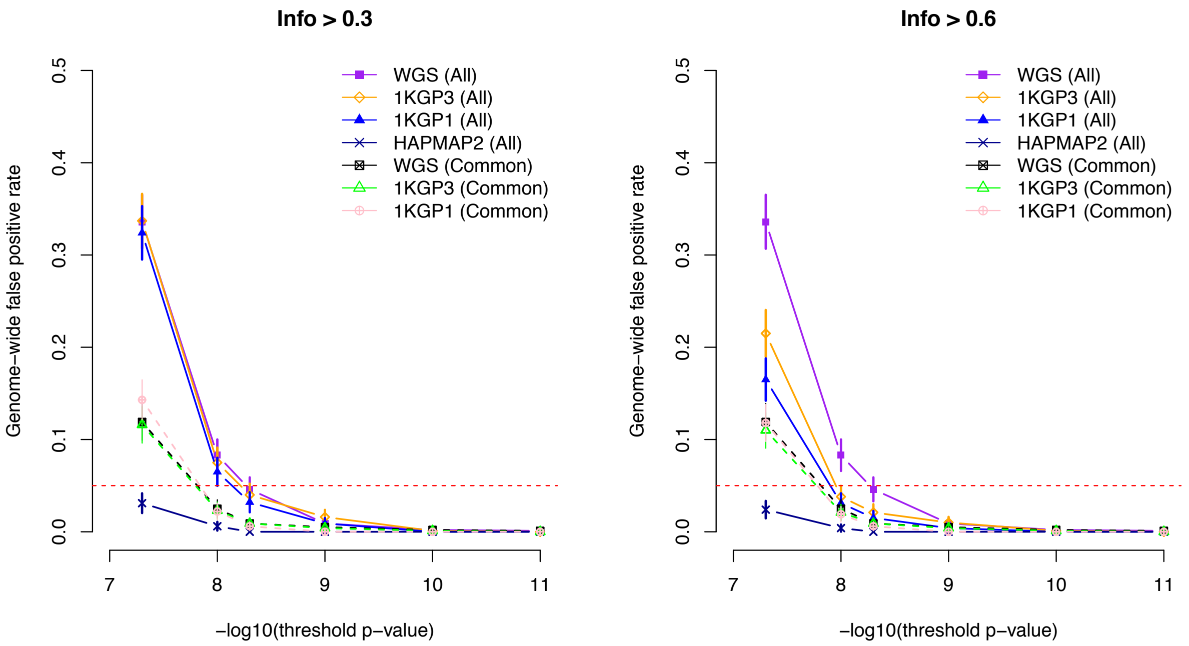

Figure S3 Genome-wide false positive rates (GWFPR) for GWAS based on different genotyping strategies at different INFO score thresholds to filter imputed SNPs. For imputed data, we removed SNPs with imputation INFO score < 0.3 (panel a) or 0.6 (panel b). The decrease in GWFPR with the increased threshold of INFO score is expected because the number of tests is smaller. Note that the false positive rate for GWAS using 1KGP-imputed SNPs at $P < 5e\text{-}8$ is still higher than expected (0.05) even if SNPs with lower INFO scores are filtered out.

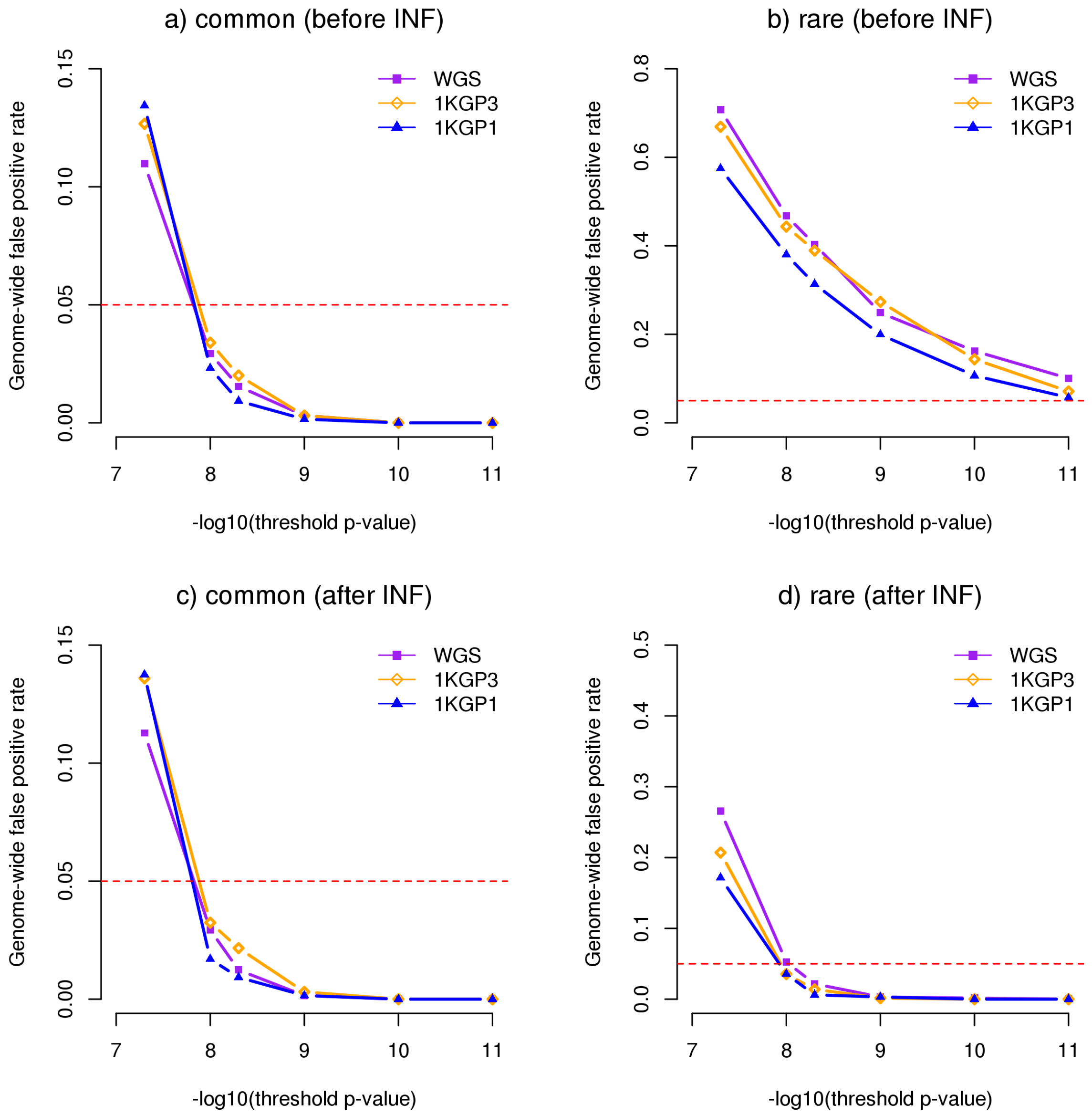

Figure S4 Inflated false positive rate (FPR) in rare variant associations due to skewed phenotype distribution under the alternative. We have shown in the figure above (Fig. S1) that FPR can be inflated if the phenotype is not normally distributed under the null that there is no genetic effect. Here, we show that even if the residuals are normally distributed, the FPR can also be inflated if there is a rare variant of relatively large effect. Shown are the genome-wide false positive rate (GWFPR, total number of simulations with at least one false positive divided by the total number of simulations) before and after inverse normal transformation (INF), at a range of *P*-value thresholds. The phenotypes were simulated under an alternative hypothesis (q^2 = 0.02) using variants on chromosome 22, and the GWAS analysis was performed for variants on chromosomes 1 to 21. The result has been adjusted for the length of genome, i.e. adjusted GWFPR = GWFPR * length (genome) / length (chromosomes 1 to 21).

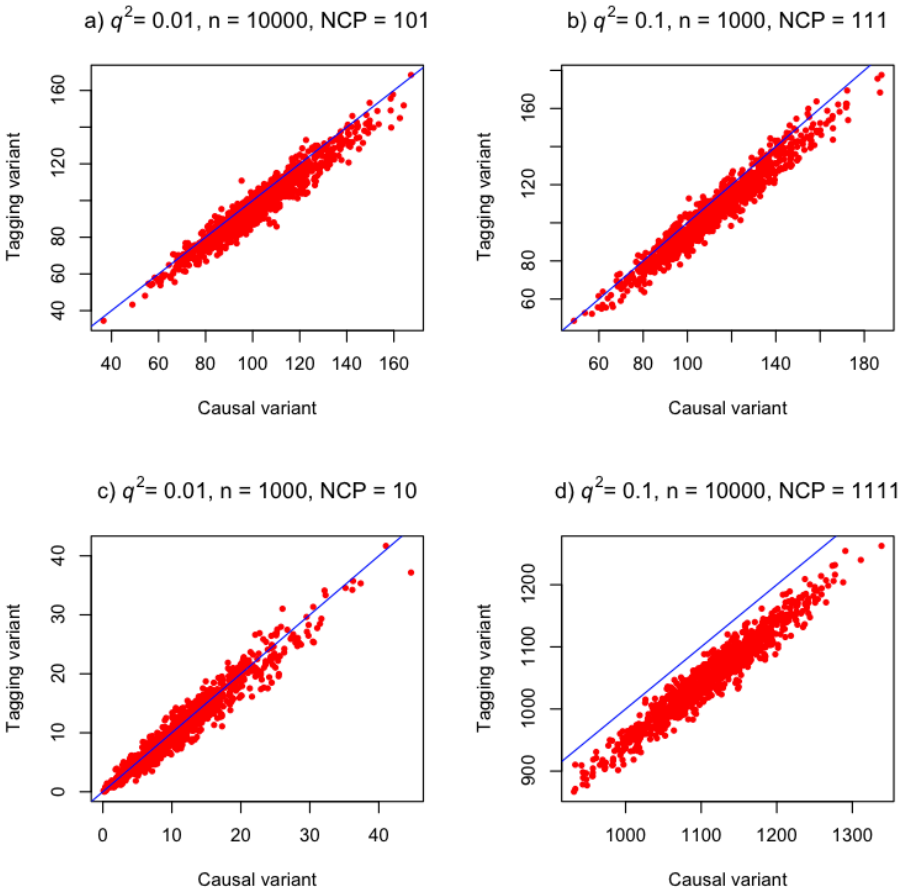

2

Figure S5 χ^2 test-statistics of the causal variant and its tagging variant. We simulated two genetic variants (MAF = 0.01 for both variants and r^2 = 0.95 between the two variants) from a bivariate binomial distribution with n = 1,000 or 10,000. We then simulated a phenotype using one of the SNPs as the causal variant with q^2 = 0.01 or 0.1. The simulations were repeated 1,000 times. Each dot represents the χ^2 statistic from association analysis of the simulated phenotype for the causal variant plotted against that for the tagging variant (the variant in LD with the causal variant). The dots above the diagonal lines represent the cases where the tagging variant other than the causal variant was detected as the top associated signal in GWAS. These results demonstrate that mapping precision of GWAS depends on NCP, which is a function of both n and q^2.

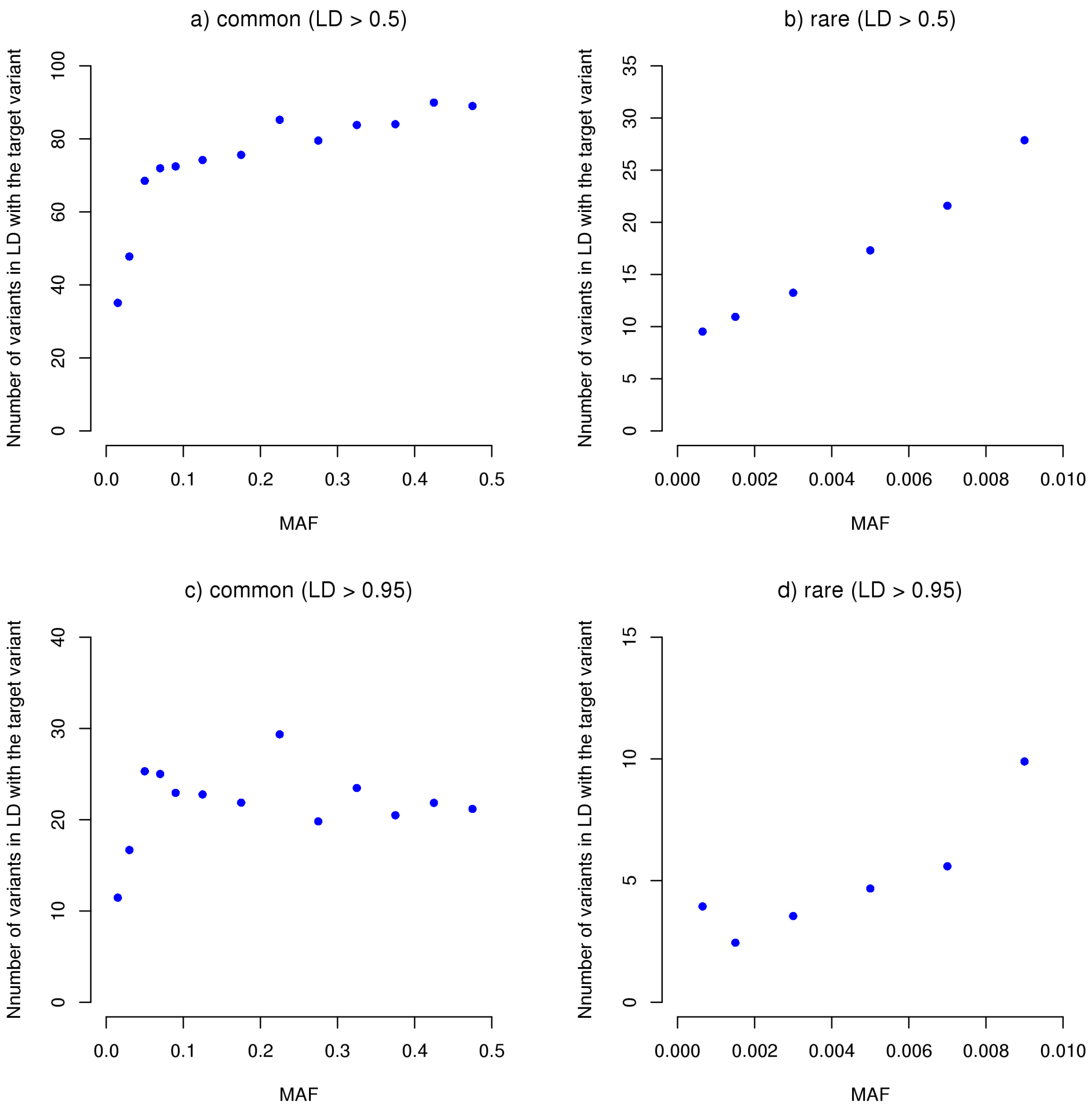

Figure S6 Number of variants in LD with a target variant as a function of MAF. We randomly sampled 100,000 as target variants from the UK10K-WGS data. We then used GCTA (the --ld option) to calculate the number of variants in LD ($r^2 > 0.5$ or 0.95) with each target variant within 100Kb distance. Each dot represents the average number in a MAF bin.

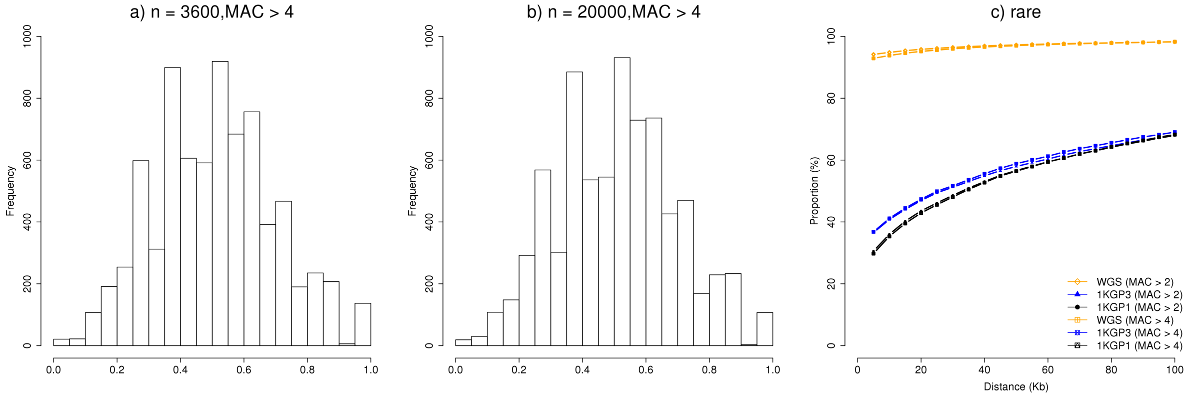

Figure S7 Sampling variation in LD r^2 and mapping precision at different thresholds of minor allele count (MAC). Shown in panels (a) and (b) is an example generated from simulations (simulating two rare variants with expected r^2 value of ~0.45), which demonstrates that the distribution of LD r^2 in a sample of 20,000 individuals is almost identical to that in a sample of 3,600 individuals at the same MAC threshold. This suggests that the sampling variation of LD r^2 depends on MAC, which is a product of MAF and sample size. We investigated the extent to which our result was affected by the sampling variation in LD by re-calculating the mapping precision for rare variants with MAC > 4 in the UK10K data. The result remained almost exactly the same (panel c), suggesting that the low mapping precision of GWAS for rare variants using imputed data is not driven by the sampling variation in LD.

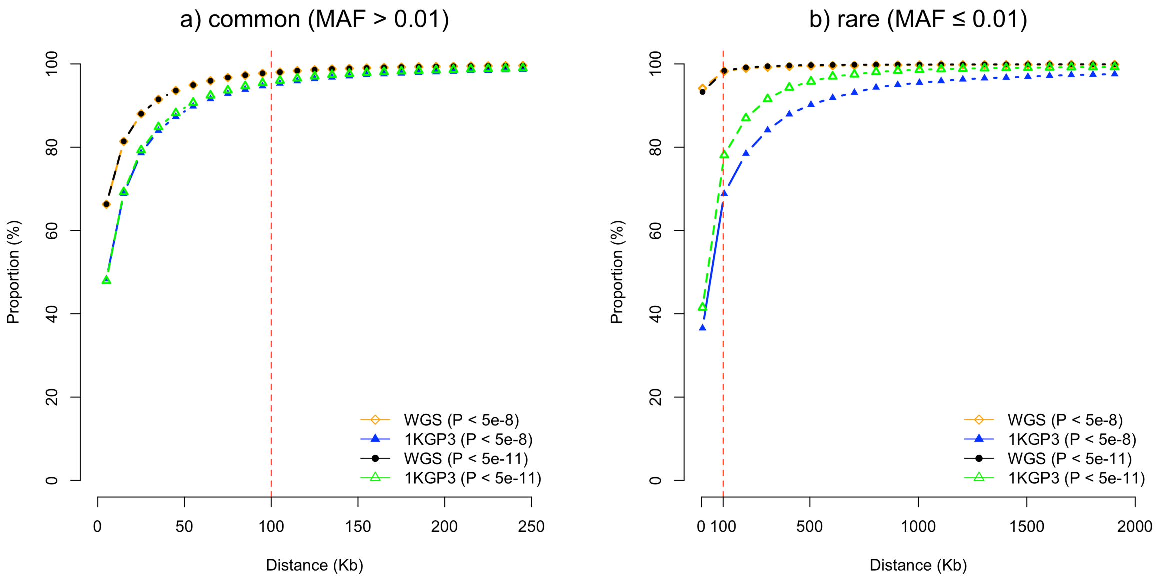

Figure S8 Extended mapping precision plot. The plotted values are the same as those in Figure 2 except that we added the results at $P < 5e\text{-}11$ and extended the *x*-axis to 250Kb for common variants and 2Mb for rare variants.

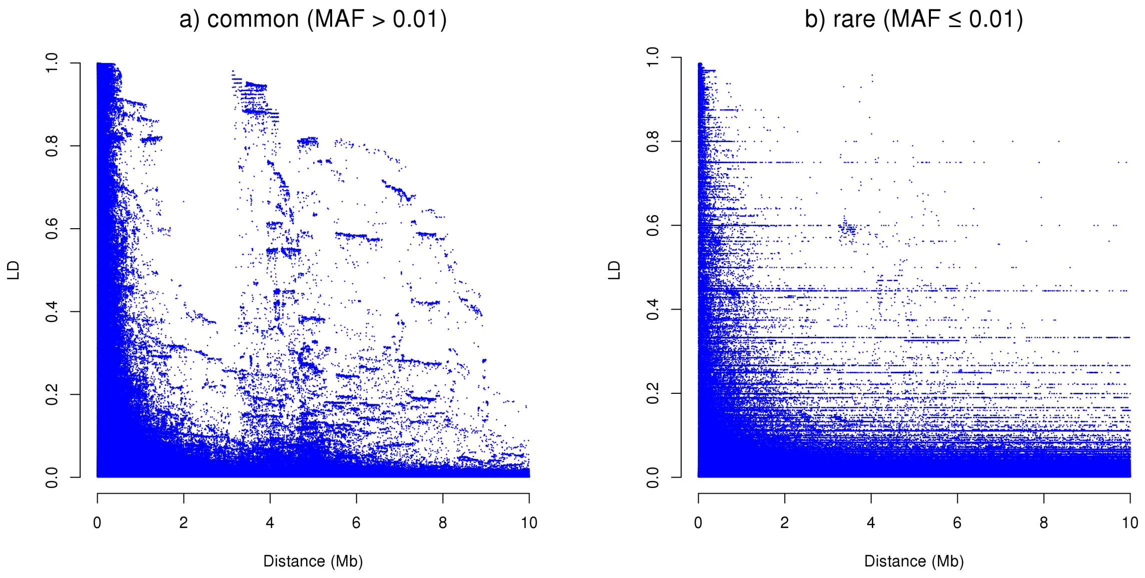

Figure S9 LD r^2 between two variants vs. their physical distance. Results are from 2,000 common and 2,000 rare variants randomly sampled from all the simulated causal variants in the UK10K-WGS data (see main text for details of the simulations). The LD r^2 was calculated between the target variant and all the other variants within 10Mb distance. The main long-range LD regions are 44.5–50.5Mb on chromosome 5 and 33–40Mb on chromosome 12 for common variants, and 43–50Mb on chromosome 8 and 89–97.5Mb on chromosome 3 for rare variants, consistent with those reported in the Price et al. study [1] (identified in European samples).

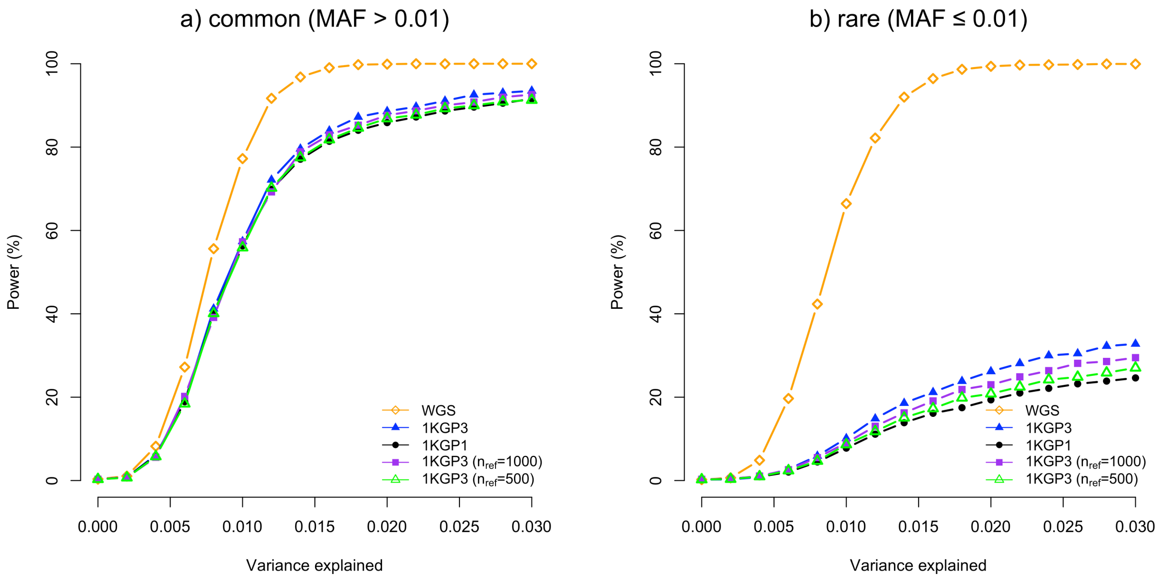

Figure S10 Statistical power of GWAS based on imputations with different reference sample sizes. Power is calculated as the proportion of simulations with a least a variant at P < 5e-8. Shown are results from 5,000 simulations for common and rare variants respectively. 1KGP3 (n_{ref} = 1000) and 1KGP3 (n_{ref} = 500): SNP array data imputed to a random subset of 1,000 and 500 individuals respectively from 1KGP3.

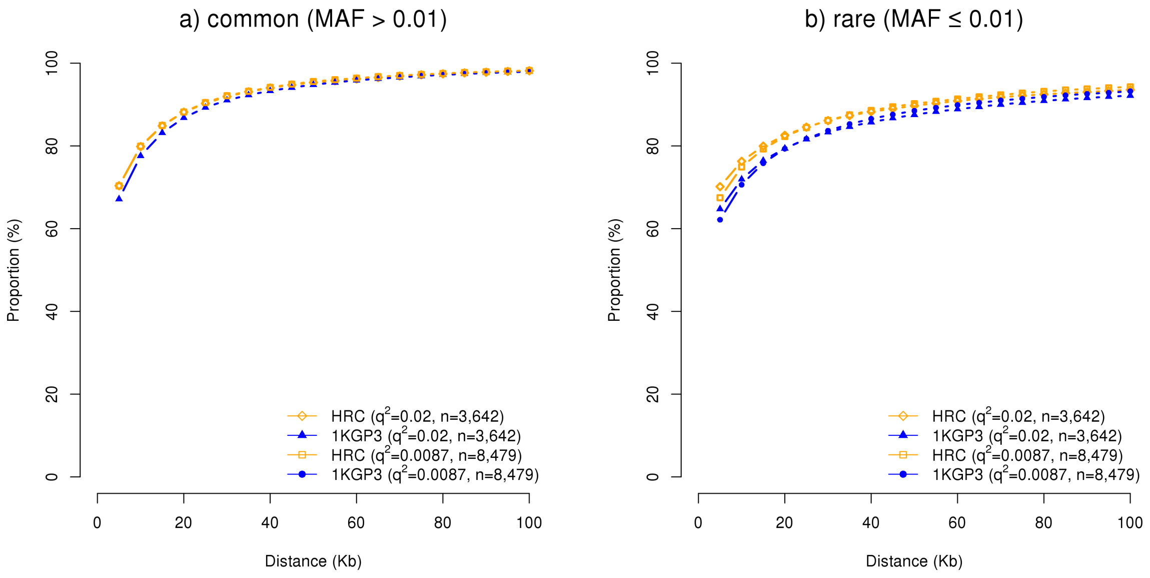

Figure S11 Mapping precision of GWAS using data from 1KGP3- and HRC-imputation in the HRS cohort. There are 8,479 unrelated individuals genotyped on ~1.7 million SNPs (1,451,882 common and 243,548 rare) in the HRS cohort. We left out 50,000 common and 50,000 rare SNPs as a pool to sample causal variants for simulations and imputed the genotypes of the remaining SNPs to 1KGP3 and HRC using the Sanger imputation server (https://imputation.sanger.ac.uk/). Shown are the results from 50,000 simulations for common (a) and rare (b) variants respectively. In each simulation replicate, we randomly sampled a variant from the causal variant pool (genotyped SNPs) and simulated a quantitative phenotype based on the method described in the main text with q^2 = 0.87% in the whole sample and q^2 = 2% in a subset of the sample (n = 3,642), and analyzed the phenotype using imputed data.

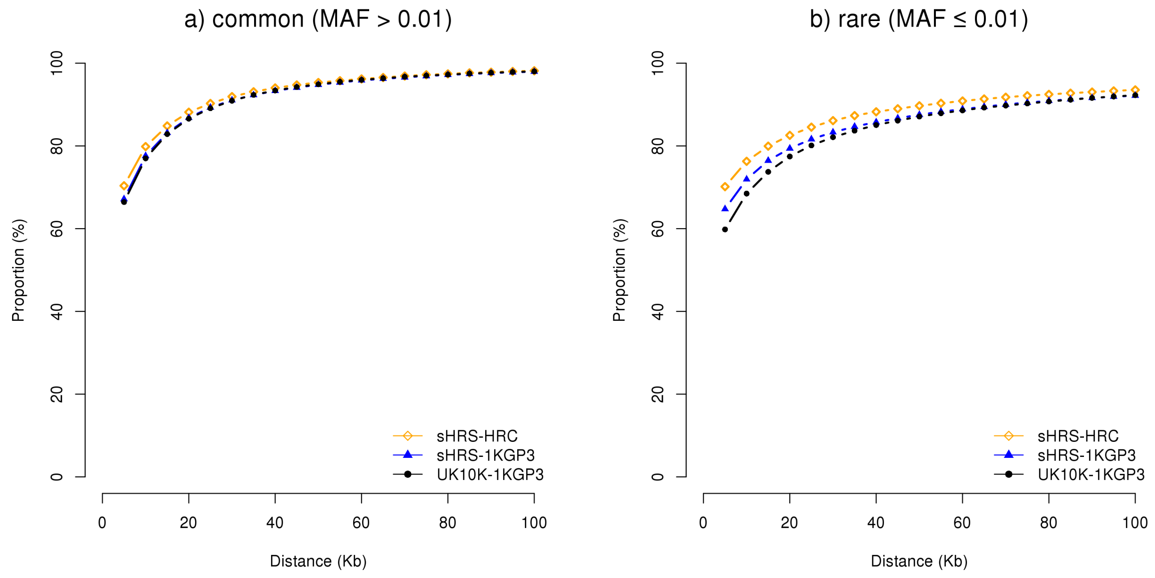

Figure S12 Mapping precision of GWAS using data from 1KGP3- and HRC-imputation in the HRS and UK10K data sets. Shown are the results from 50,000 simulations with $q^2 = 2\%$. sHRS-HRC: a subset of the HRS genotype data (n = 3,642) imputed to HRC (n_{ref} = 32,488). sHRS-1KGP3: a subset of the HRS genotype data (n = 3,642) imputed to 1KGP3 (n_{ref} = 2,504). UK10K-1KGP3: UK10K 'array data' (n = 3,642) imputed to 1KGP3 (n_{ref} = 2,504). In the UK10K simulation, mapping precision was calculated focusing only on causal variants that exist in the 1KPG3 data.

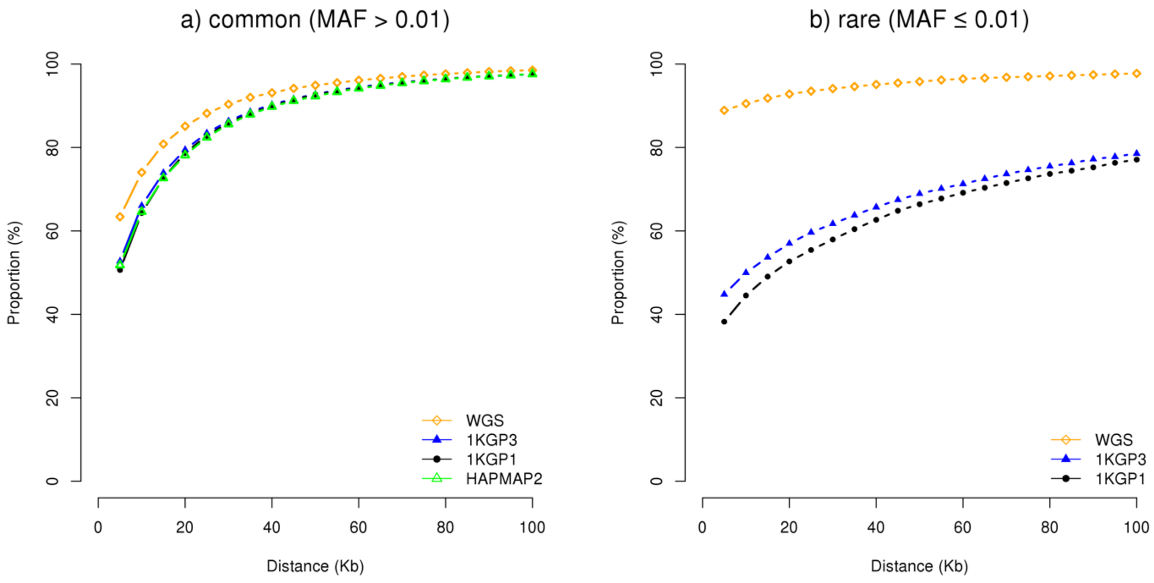

Figure S13 Mapping precision of GWAS based on different genotyping strategies observed from simulations where the causal variants are sampled from variants available in the imputation references. Shown on the *y*-axis is the proportion of causal variants that were mapped to variants within a certain distance as specified on the *x*-axis.

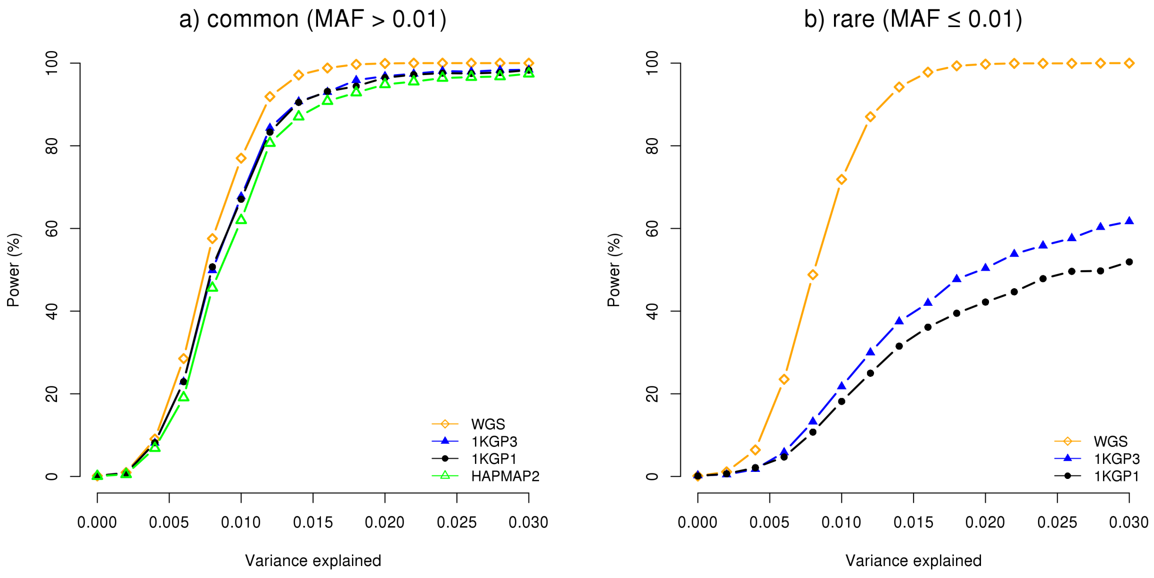

Figure S14 Statistical power of GWAS based on different genotyping strategies observed from simulations where the causal variants are sampled from variants available in the imputation references. Power is calculated as the proportion of simulations with a least a variant at $P < 5e\text{-}8$. Shown are the results from 5,000 simulations for common and rare variants respectively at each heritability level.

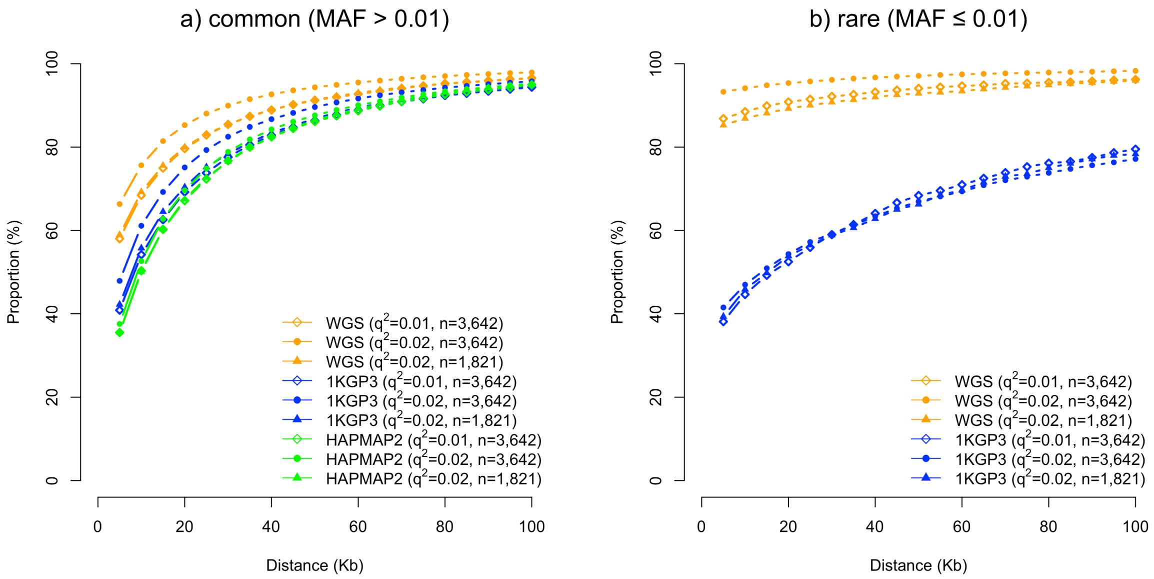

Figure S15 Mapping precision of GWAS with different sample sizes and q^2. The analyses were performed using the same method as used in Figure 2 but with different sample sizes and q^2 values.

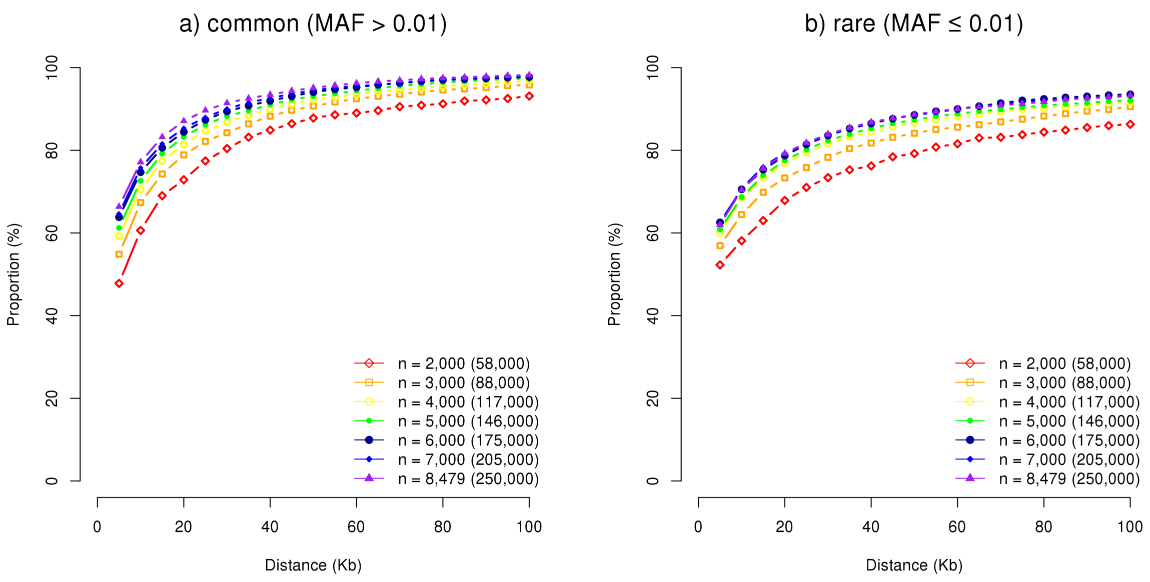

Figure 16 Mapping precision increases with the increase of GWAS sample size. The analyses were performed in the genotyping data from the Health Retirement Study (HRS). The results are from 10,000 simulation replicates for common and rare variants respectively. In each simulation replicate, we randomly sampled a variant from the causal variant pool (50,000 each) and simulated a quantitative phenotype based on the method described in the main text with q^2 = 0.87% (NCP = ~74), and analyzed the phenotype using imputed data with a range of sample sizes shown on the legend. The numbers in the parentheses are the equivalent GWAS sample size if q^2 = 0.03%.

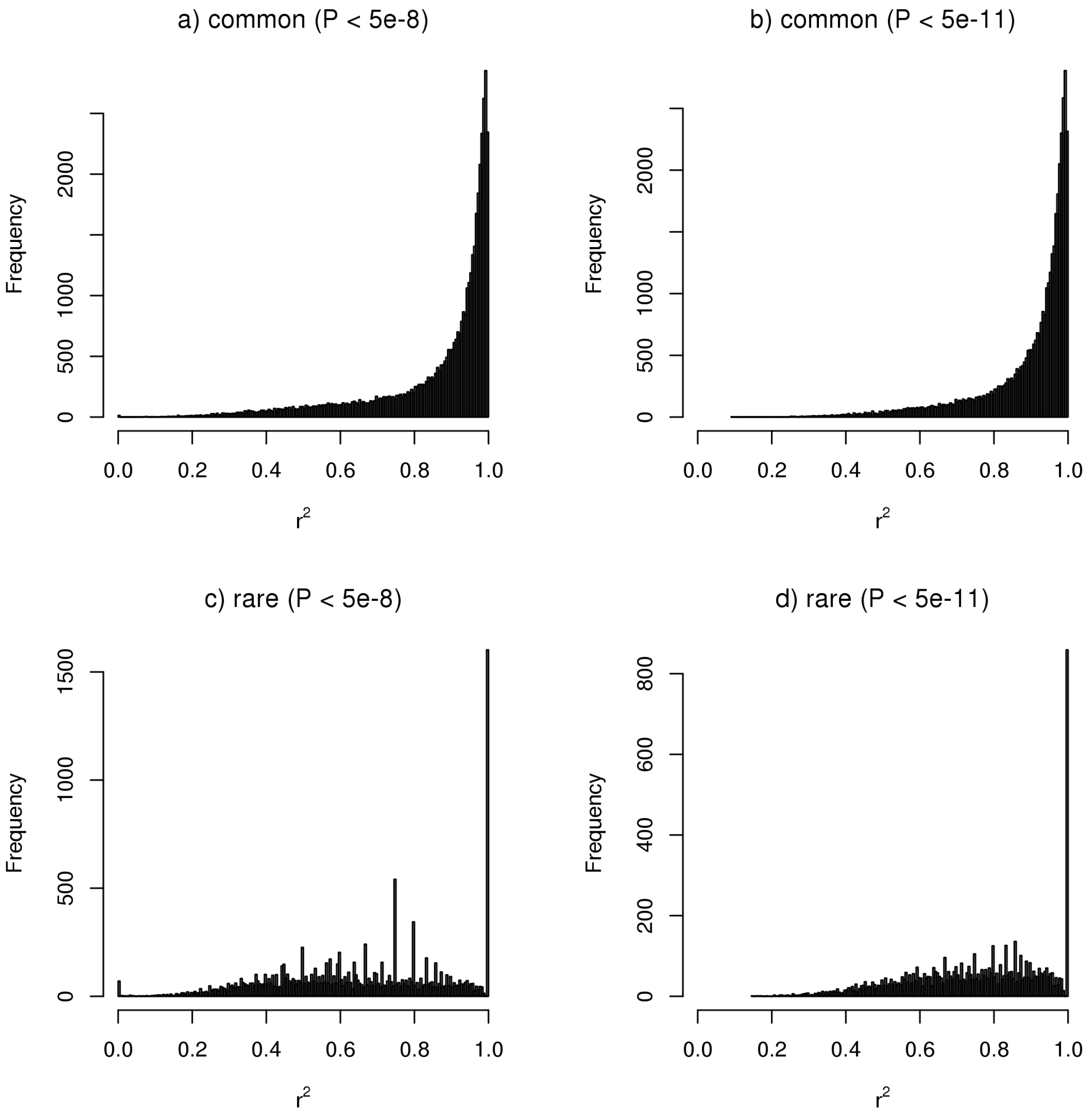

Figure S17 Distribution of squared correlations between WGS and 1KGP3-imputed data at different *P*-value thresholds. Shown is squared correlation between the genotype of the causal variant from WGS and the best-guess genotype of the GWAS hit (at *P* < 5e-8 or 5e-11) from the 1KGP3-imputed data.

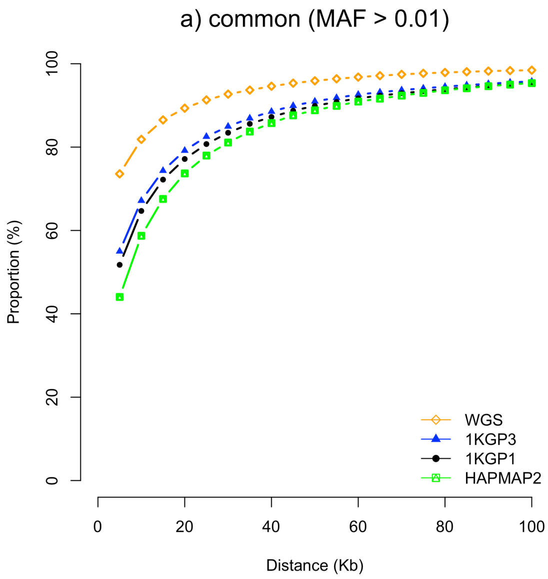

Figure S18 Mapping precision of GWAS with different genotyping strategies when the causal variants are all at DHS.

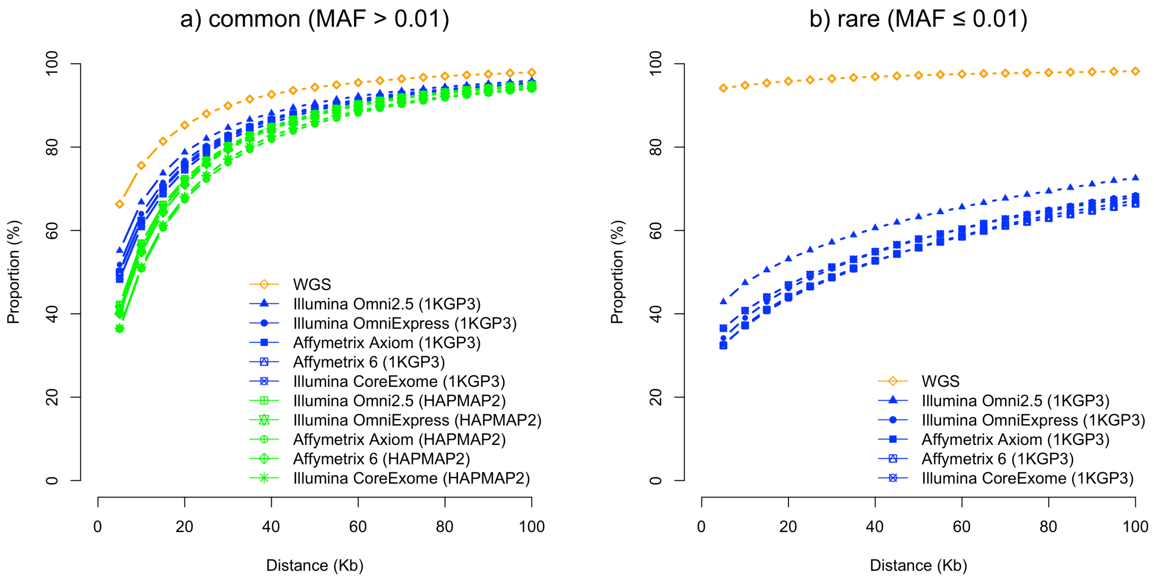

Figure S19 Mapping precision of GWAS using imputed data based on different SNP genotyping arrays. The analyses were performed using the same method as used in Figure 2 but with different SNP genotyping arrays. Illu2M: Illumina Omni2.5; illu1M: Illumina OmniExpress; affy6: Affymetrix 6; affyAxiom: Affymetrix Axiom Genome-Wide EUR Array; coreExome: Illumina CoreExome.

Table S1 Number of variants for each of the genotyping strategies after quality control.

**Genotyping strategy**	Number of common variants	Number of rare variants
UK10K-WGS	8,325,271	9,287,442
1KGP3	9,351,166	10,669,647
1KGP1	8,897,418	7,841,795
1KGP3-1000	9,291,256	10,037,982
1KGP3-500	9,238,091	9,086,824
HapMap2	2,394,254	96,854

Common variants: MAF > 0.01; Rare variants: MAF ≤ 0.01.

Table S2 Genomic inflation factors from 1000 simulations under the null model.

**Genotyping strategy**	Genomic inflation factors
UK10K-WGS	1.0006
1KGP3	1.0006
1KGP1	1.0007
HapMap2	1.0006

Table S3 Number of genome-wide significant top variants for each of the genotyping strategies in 50,000 simulations. Genome-wide significant: p-value < 5e-8.

**Genotyping strategy**	Number of significant common variants	Number of significant rare variants
UK10K-WGS	49,976	49,627
1KGP3	44,376	13,381
1KGP1	43,016	9,978
1KGP3-1000	43,733	11,920
1KGP3-500	43,228	10,884
HapMap2	38,359	

Common variants: MAF > 0.01; Rare variants: MAF ≤ 0.01.

Note that the purpose of this analysis is to quantify the proportion of genome-wide significant top variants within a given physical distance of the corresponding causal variants. We therefore assigned a relatively large effect size to the simulated causal variant so that the variance explained by each causal variant (q^2) was 2%. The expected chi-squared value for the causal variant is ~75 given $q^2 = 2\%$ and $n = 3{,}642$, which is much larger than the genome-wide significant level (corresponding to a chi-squared value of about 30). This explains why the power for GWAS using UK10K-WGS data is almost 100% (first row of the table).

Table S4 Numbers of variants in UK10K-WGS in common with those in five SNP genotyping arrays used in simulations.

**SNP array**	# Variants
Illumina CoreExome	312,264
Affymetrix 6.0	572,172
Affymetrix Axiom Genome-Wide EUR Array	573,241
Illumina OmniExpress	607,344
Illumina Omni2.5	1,540,717

Text S1: Acknowledgments

UK10K (EGA **accessions: EGAS00001000108 and EGAS00001000090):** The UK10K project was funded by the Wellcome Trust award WT091310. Twins UK (TUK): TUK was funded by the Wellcome Trust and ENGAGE project grant agreement HEALTH-F4- 2007–201413. The study also receives support from the Department of Health via the National Institute for Health Research (NIHR)-funded BioResource, Clinical Research Facility and Biomedical Research Centre based at Guy's and St. Thomas' NHS Foundation Trust in partnership with King's College London. Dr Spector is an NIHR senior Investigator and ERC Senior Researcher. Funding for the project was also provided by the British Heart Foundation grant PG/12/38/29615 (Dr Jamshidi). A full list of the investigators who contributed to the UK10K sequencing is available from http://www.UK10K.org.

HRS (dbGaP accession: phs000428.v1.p1): HRS is supported by the National Institute on Aging (NIA U01AG009740). The genotyping was funded separately by the National Institute on Aging (RC2 AG036495, RC4 AG039029). Genotyping was conducted by the NIH Center for Inherited Disease Research (CIDR) at Johns Hopkins University. Genotyping quality control and final preparation of the data were performed by the Genetics Coordinating Center at the University of Washington.

Reference

1. Price AL, Weale ME, Patterson N, Myers SR, Need AC, Shianna KV, Ge D, Rotter JI, Torres E, Taylor Kent D, et al: **Long-Range LD Can Confound Genome Scans in Admixed Populations.** *Am J Hum Genet* 2008, **83:**132-135.
